# Synthesis and Cytocompatibility of Bacterial Nanocellulose–Hydroxyapatite Biocomposites with Mg, Cu, Zn, and Sr Substitution

**DOI:** 10.1021/acsomega.6c04779

**Published:** 2026-09-21

**Authors:** Michele C. Formolo, Bruna Segat, Sandro R. K. Junior, Karina Cesca, Andrea L. dos S. Schneider, Ana P. T. Pezzin, Luismar M. Porto

**Affiliations:** † Department of Chemical Engineering, University of the Joinville Region, Joinville 89219-710, Brazil; ‡ Postgraduate Program in Health and Environment, University of the Joinville Region, Joinville 89219-710, Brazil; § Postgraduate Program in Process Engineering, University of the Joinville Region, Joinville 89219-710, Brazil; ∥ Program in Food Engineering, 28117 Federal University of Santa Catarina, Florianópolis 88040-900, Brazil; ⊥ Postgraduate Program in Chemical Engineering, Federal University of Santa Catarina, Florianópolis 88040-900, Brazil

## Abstract

Bacterial nanocellulose (BNC) combined with hydroxyapatite (HAp) forms biocomposites that exhibit biomimetic properties favorable for bone regeneration. Moreover, incorporating trace elements naturally present in bone, such as Mg, Cu, Zn, and Sr, has been reported to enhance osteogenic activity and support vascularization. Despite these advantages, BNC’s resistance to enzymatic degradation in vivo remains a critical limitation. To address this, BNC-based biocomposites containing cation-substituted hydroxyapatite (at varying concentrations of Mg^2+^, Cu^2+^, Zn^2+^, or Sr^2+^) were synthesized and bioactivated in simulated body fluid (SBF). These materials were characterized using scanning electron microscopy/energy dispersive spectroscopy (SEM/EDS), Fourier transform infrared/attenuated total reflectance (FTIR/ATR), X-ray diffraction (XRD), thermogravimetric analysis (TGA) and in vitro cytotoxicity assays. The most promising compositions were further evaluated for osteoblast (MC3T3-E1) adhesion and enzymatic biodegradation. SEM confirmed the uniform distribution of HAp crystals across the BNC network, while TGA revealed a high residual mass (51.9–65.6%), indicating effective HAp incorporation. Cytotoxicity results showed that the BNC/ZnHAp 1% and BNC/SrHAp 1% samples maintained cellular metabolic activity at approximately 90%, indicating biocompatibility. Additionally, these biocomposites promoted superior cell proliferation compared to the nonsubstituted BNC/HAp control at all time points. Overall, the incorporation of selected cations enhanced the biological performance of BNC/HAp biocomposites, suggesting their suitability for further investigation in bone tissue repair and implantable biomedical applications.

## Graphical abstract

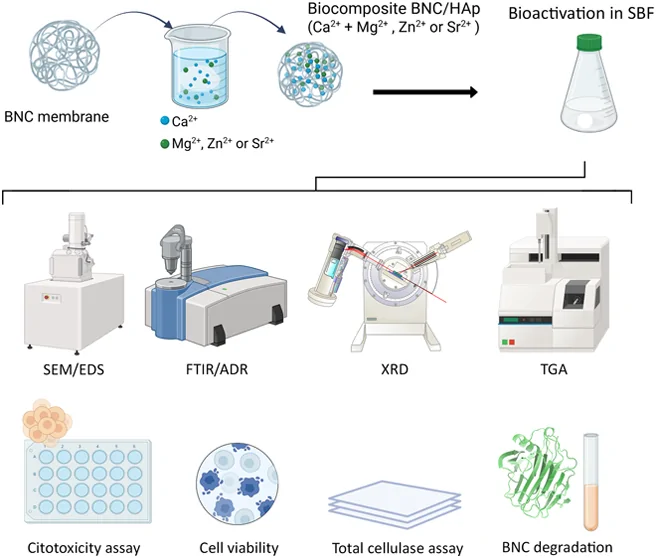

## 1 Introduction

Traumas caused by accidents or degenerative diseases can cause bone defects, affecting people’s quality of life and frequently demanding surgical intervention. The most common practices in these cases include autologous grafts, allografts, hybrid grafts, demineralized heterologous bone, the induced membrane technique and other materials used as bone substitutes.^1,2^ Bone tissue engineering (BTE) is an innovative approach to solving this problem. The principle behind these techniques involves creating an isolated space with a barrier membrane between the soft tissue and the bone defect, preventing the invasion of rapidly growing fibroblasts and epithelial cells into bone defects, thus allowing time for osteogenic cells to promote bone tissue growth.^3^ This method overcomes several limitations of traditional treatments, such as inflammation, poor osseointegration and immunological rejection.^4,5^

Materials such as three-dimensional (3D) scaffolds play an essential role in this process because they provide a physical support during the regeneration of damaged tissues.^6^ It is fundamental to note that in a living organism, the cellular microenvironment is inherently three-dimensional. This complex and dynamic biological environment, represented by the Extracellular Matrix (ECM), plays an essential role in cell development and behavior, being involved in important processes such as regulation of cell functions, growth responses, migration and the production of new bone by osteoblasts and osteocytes.^7^ To replicate ECM, the development of 3D scaffolds should serve as a substrate for cell interactions, sustain newly grown tissue, provide nutrients and stimulate cell invasion.^8^

Hydrogels, composed of chemically or physically cross-linked hydrophilic polymeric networks, represent an ideal scaffold for mimicking the ECM because of their biocompatibility, tissue adhesion and biomimetic design, among others.^9^ In this sense, bacterial nanocellulose (BNC) hydrogels stand out for their nanostructure and morphology similar to collagen, enabling BNC to mimic collagen fibrils in the ECM of connective tissues.^10,11^ Bacterial nanocellulose is a natural polysaccharide produced by several species of bacteria,^11,12^ and it has important properties for biomedical applications, such as biocompatibility, high water retention capacity, high porosity, compatibility with living tissues, and nontoxicity and nonallergenicity.^13^ Despite this, BNC alone is not a bioactive material. So, several studies aim to improve the physical properties and promote bioactivity and osteogenic activity in BNC.^12^ Studies have been carried out with the aim of producing BNC biocomposites with hydroxyapatite (HAp), a naturally occurring bone component, to overcome the disadvantages of each one alone,^12,14–16^ mainly by stimulating the adhesion and proliferation of osteoblasts.^17^

Furthermore, bone tissue is naturally a composite, composed not only of calcium phosphate but also other elements such as Na^+^, Mg^2+^ or Sr^2+^ in the form of trace elements. These trace elements are vital in bone growth and repair. They are able to control degradation, increase the mechanical strength of materials, and regulate their bioactive properties, such as osteoconduction.^18,19^ Thus, studies have shown that replacing Ca and P sites in HAp can increase degradation kinetics and also promote even more cell growth, therefore improving the interface between bioceramics and biological tissue.^18,20,21^

However, the lack of biochemical processes capable of facilitating the in vivo biodegradability of BNC, hereby rendering it a truly bioabsorbable biomaterial, represents an obstacle to be addressed.^22–24^ Seeking solutions, some research has been carried out toward partial preoxidation using chemical agents such as sodium periodate,^25^ TEMPO (2,2,6,6-tetrametylpiperidine-1-oxyl)^26^ or using 2,3-dialdehyde bacterial cellulose as an alternative to improve the degradability of BNC, however, it is essential to highlight that chemical modification damages the mechanical properties of BNC, which might limit its application for specific tissue repair.^22,27^

In addition to the investigation of the enzymatic degradation of regenerated cellulose (RCS) by cellulases and lysozyme,^28^ cellulases are known to rapidly degrade cellulose under appropriate conditions.^29^ These enzymes are generally produced by fungi such as *Trichoderma sp*., *Aspergillus sp*. and *Penicillium sp*., with optimal activity around pH 5 for cellulase^30,31^ and pH 6.5 for lysozyme.^32^ However, when considering the metabolic processes involved in bone regeneration, in which alkaline phosphatase acts as the main biochemical marker, with ideal activity between pH 8.5 and 9.5,^33^ and the physiological pH of the human body, approximately 7.4,^34^ cellulase activity can be inhibited at these pH values.

Therefore, this study aimed to develop and characterize BNC biocomposites incorporating HAp and HAp with replacement of Ca by Mg^2+^, Cu^2+^, Zn^2+^, and Sr^2+^, and to bioactivate them in simulated body fluid (SBF). The most promising samples from each substitution group that do not exhibit cytotoxic effects will be further evaluated for cellulase-mediated enzymatic degradation and cell proliferation, with the objective of assessing their potential as implantable biomaterials.

## 2 Materials and Methods

### 2.1 Synthesis and Purification of BNC Membranes

The production of BNC membranes was carried out by the bacterium *Komagataeibacter hansenii* (ATCC 23769). The culture medium for cell reactivation and membrane production was Mannitol Medium, consisting of 20.0 g L^–1^ of mannitol, 5.0 g L^–1^ of peptone and 5.0 g L^–1^ of yeast extract. The cells were reactivated in an Erlenmeyer flask and incubated at 30 °C under static conditions for 2 days. Then, this preinoculum was transferred to the culture medium at a 20% (v/v) concentration, with an optical density between 0.15 and 0.19, measured by absorbance at 600 nm in a spectrophotometer. The cultivation was carried out in Erlenmeyer flasks under static conditions at 30.0 °C for 9 days. The BNC membranes formed on the surface of the culture medium were removed, washed, and treated with 0.1 mol/L of NaOH at 80.0 °C for 60 min in a thermostatic bath. Afterward, the membranes were washed with distilled water until reaching pH 7.0, autoclaved and stored in a refrigerator.^16^

### 2.2 Production of Biocomposites and Bioactivation in Simulated Body Fluid

The biocomposites were produced using the immersion-cycle technique, following the methodology of Hutchens et al.^35^ Initially, the samples were submerged in 20.0 mL of solutions containing either CaCl_2_ or CaCl_2_ combined with MgCl_2_, CuCl_2_, ZnCl_2_, or SrCl_2_. These immersions lasted 24 h under constant agitation in an orbital shaker, maintained at 26.0 °C and 85 rpm. The dopant concentrations of MgCl_2_, CuCl_2_, ZnCl_2_, and SrCl_2_ were prepared at 1%, 3%, and 5% molar, as detailed in Table 1. After each immersion, the membranes were rinsed to eliminate excess reagents and transferred into a 0.06 M Na_2_HPO_4_ solution for another 24 h. This procedure was repeated for a total of three complete immersion cycles to increase the mineral deposition in the biocomposites.^35^ All solutions were prepared to maintain a stoichiometric hydroxyapatite ratio (Ca/P = 1.67). In the doped formulations, the ratio was calculated as (Ca + M)/P = 1.67, where M corresponds to the dopant ion. The choice for using such low concentrations of ions is because other studies of our group tested higher concentrations of MgCl_2_ and CuCl_2_, and in the cytotoxicity analysis, all samples doped with copper were cytotoxic, and only one sample with Mg did not show a cytotoxic effect.^16^

**Table 1:** Concentrations of Mg^2+^, Cu^2+^, Zn^2+^ and Sr^2+^ Functionalization Solutions

	Concentration (mol/L)					
Sample	[Ca^2+^]	[Mg^2+^]	[Cu^2+^]	[Zn^2+^]	[Sr^2+^]	[PO_4_^3–^]
BNC/HAp	0.1	-	-	-	-	0.06
BNC/MgAp 1%	0.099	0.001	-	-	-	
BNC/MgAp 3%	0.097	0.003	-	-	-	
BNC/MgAp 5%	0.095	0.005	-	-	-	
BNC/CuAp 1%	0.099	-	0.001	-	-	
BNC/CuAp 3%	0.097	-	0.003	-	-	
BNC/CuAp 5%	0.095	-	0.005	-	-	
BNC/ZnAp 1%	0.099	-	-	0.001	-	
BNC/ZnAp 3%	0.097	-	-	0.003	-	
BNC/ZnAp 5%	0.095	-	-	0.005	-	
BNC/SrAp 1%	0.099	-	-	-	0.001	
BNC/SrAp 3%	0.097	-	-	-	0.003	
BNC/SrAp 5%	0.095	-	-	-	0.005	

Subsequently, the biocomposites were incubated in 5.0 mL of simulated body fluid (SBF) to promote in vitro apatite formation. The SBF was prepared in accordance with Kokubo et al.^36^ and ISO 23317:2014, using the following reagents: 6.55 g NaCl, 2.27 g NaHCO_3_, 0.37 g KCl, 0.18 g Na_2_HPO_4_·2H_2_O, 0.30 g MgCl_2_·6H_2_O, 40 mL of 1 M HCl, 0.37 g CaCl_2_·2H_2_O, 0.07 g Na_2_SO_4_, and 6.06 g of TRIS, all dissolved in 700 mL of distilled water and then brought up to 1000 mL. The membranes, placed in polypropylene flasks with the SBF, were kept at 37.0 °C for 14 days. The SBF was refreshed every 4 days. After incubation, the samples were washed with distilled water, freeze-dried using a Terroni LT1000 lyophilizer, and then characterized. Figure 1 shows the methodology steps.

**Figure 1:**
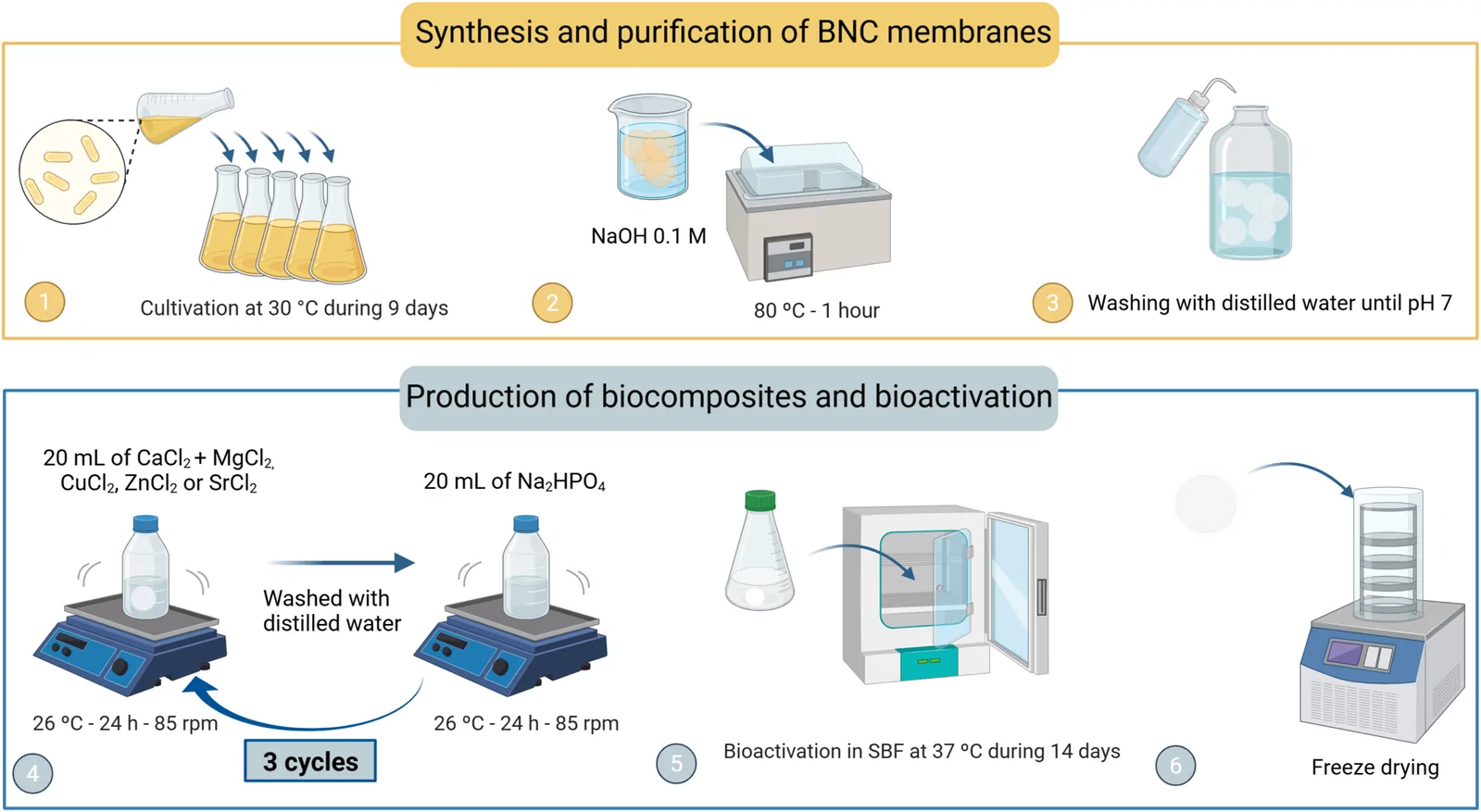
Schematic representation of BNC membranes synthesis and biocomposites production. Graphic created in BioRender.com.

### 2.3 Characterization

#### 2.3.1 Scanning Electron Microscopy with Dispersive Energy Spectroscopy (SEM/EDS)

Surface morphology and elemental composition of the samples were examined using a JEOL JSM-6390LV scanning electron microscope (JEOL, Tokyo, Japan), equipped with a tungsten filament. The analyses were performed at an accelerating voltage of 10 kV.

#### 2.3.2 Fourier Transform Infrared Spectroscopy with Attenuated Total Reflectance (FTIR/ATR)

FTIR analyses were carried out on a PerkinElmer Spectrum spectrometer using the attenuated total reflectance (ATR) module. Each spectrum was acquired with 32 scans per sample, within the spectral range of 4000 to 500 cm^–1^, and a resolution of 4 cm^–1^.

#### 2.3.3 Wide-Angle X-ray Diffractometry (XRD)

X-ray diffraction was measured using a Bruker XRD 8 Advance diffractometer. The measurements were performed with Cu Kα radiation (λ = 1.5418 Å), operated at 40 kV and 25 mA (total power: 1000 W), and equipped with a 160-channel digital detector. The scanning range was set from 5° to 90° in 2θ, with a step size of 0.02°/s.

#### 2.3.4 Thermogravimetric Analysis (TGA)

Thermal stability and the organic/inorganic content of the biocomposites were determined by thermogravimetric analysis using a TGA-Q50 analyzer (TA Instruments). Samples weighing between 5 and 10 mg were heated from 25 to 1000 °C at a rate of 10 °C/min under an oxidizing atmosphere. This analysis was also used to assess the degree of mineral incorporation into the membrane matrix.

#### 2.3.5 Cytotoxicity Assay

The cytotoxic potential of the samples was assessed by evaluating the metabolic activity of L929 mouse fibroblast cells, following the ISO 10993–5:2009. Mouse fibroblast cells (L929) were maintained in Dulbecco’s Modified Eagle’s Medium (DMEM) medium supplied with 10% FBS and 1% antibiotics. Cells were cultured in an incubator with 5% CO_2_ at 37 °C and 95% humidified air during the in vitro tests. In vitro cytotoxicity of the sample was performed through the direct contact method. The L929 cells were seeded in 24-well culture plates in 4.0 × 10^4^ cells/well (Corning Life Sciences). After 24 h, samples were added. Cells were incubated for 24 h at 37 °C and 5% CO_2_. After each cultivation time, the culture medium and samples were removed. Then, the cells were washed with PBS, and cell viability was measured using an MTS assay.^37^ Then, the remaining medium was transferred into 96 well plates to measure optical density at a wavelength of 490 nm using a spectrophotometer (Molecular Devices). The metabolic activity was calculated concerning the sample control. Data from in vitro cell culture were statistically analyzed using one-way repeated Analysis of Variance (ANOVA), followed by Tukey’s post hoc test for multiple comparisons. Statistical significance was considered at a confidence level of α = 0.05. The data represents the mean ± standard error from three independent assays in triplicate.

#### 2.3.6 Cell Viability and Proliferation Assay

MC3T3-E1 osteoblastic cells were used to evaluate cell viability and proliferation on the biocomposite surfaces, following the methodology described by dos Santos et al.^38^ Cells were cultured in α-MEM medium (α-Modified Eagle’s Medium, M0644, Sigma) supplemented with 10% fetal bovine serum (FBS, Sigma-Aldrich) and 1% penicillin/streptomycin (Sigma-Aldrich). Upon reaching approximately 80% confluency, cells were detached using TrypLE Express Enzyme (Thermo Fisher Scientific), then centrifuged at 5000 rpm for 2 min to eliminate the enzyme. The harvested cells were resuspended in fresh medium and counted using a Neubauer chamber (SP Labor, Brazil).

Sterilized biocomposites were placed in 96-well tissue culture plates and prehydrated with culture medium for 24 h. After this incubation period, the medium was discarded, and osteoblasts were seeded at a density of 1.5 × 10^5^ cells per cm^2^ onto each biomaterial. Biocomposites containing BNC/HAp served as controls. The assay was carried out over 21 days, with evaluations conducted on days 1, 7, 14, and 21. At each time point, samples were rinsed with PBS, and the MTS assay was employed to measure the metabolic activity of adherent cells.

This experiment was performed in octuplicate and included only BNC/HAp, BNC/MgAp, BNC/ZnAp, and BNC/SrAp biocomposites at a 1% concentration, these being the compositions that exhibited the least cytotoxic impact on cell metabolism. The resulting cell proliferation data were statistically analyzed. First, the Shapiro-Wilk test assessed normality at a 0.05 significance level (95% confidence). A p-value below 0.05 indicates a non-normal (nonparametric) data distribution, which implies that traditional parametric methods are not applicable.

For data sets that did not follow a normal distribution, the Kruskal–Wallis test was used at a 95% confidence level (*p* < 0.05). As explained by Chicco, Sichenze and Jurman (2025)^39^ this test is suitable for comparing more than two independent groups. The hypotheses tested included the null hypothesis (no significant difference among the dopants) and the alternative hypothesis (a significant difference). If *p* > 0.05, the differences were considered statistically insignificant; if *p* < 0.05, the differences were considered statistically significant. The Mann–Whitney *U* test was used to compare each doped sample further with the calcium-based control.

#### 2.3.7 Total Cellulase Assay

In line with the method described by Zhang et al.,^40^ total cellulase activity was assessed, taking into accounting for the activities of endoglucanases, exoglucanases, and β-d-glucosidases. For this purpose, enzyme activity was evaluated using insoluble cellulose substrates such as Whatman No. 1 filter paper, in accordance with the modified filter paper assay (FPA) protocol.^40,41^ Strips of Whatman No. 1 filter paper (1.0 × 6.0 cm^2^, approximately 50 mg) were placed in test tubes with 1 mL of 50 mM citrate buffer (pH 4.8) and 1 mL of enzyme extract (diluted when needed). These mixtures were incubated at 50 °C for 60 min. Following incubation, 1 mL of the reaction mixture was mixed with 1 mL of DNS reagent and heated at 100 °C for 5 min. The samples were then cooled in an ice bath, and 10 mL of deionized water was added. Absorbance was measured at 540 nm using a UV–vis spectrophotometer.

A glucose standard curve ranging from 0.25 to 2.0 mg/mL was used for calibration. The absorbance values were interpolated to determine reducing sugar concentrations (μmol/mL). Enzyme activity was calculated using the following eq 1

1 $$\left[enzyme\right]=\left[RS\right]\times V_{mixture}\times t_{reaction}^{-1}$$

where [RS] is the concentration of reducing sugars produced (μmol·mL^–1^), *V* is the volume of the reaction mixture (mL), and *t* is the enzymatic reaction time (min). The cellulase activity determined by the FPA assay was 145.27 IU/mL, which was used to standardize the enzyme concentration used in subsequent BNC degradation experiments.

#### 2.3.8 Preliminary Study of BNC Degradation in Enzymatic Solution Evaluating the Influence of pH

The protocol adapted from Oliveira, Rambo e Porto^42^ and Stankova et al.^43^ was employed to investigate the degradation behavior of BNC in different pH environments. BNC discs (approximately 0.5 cm in diameter) were placed in test tubes containing phosphate buffer solutions at pH 7 and 8.5. After autoclaving, 100 μL of cellulase enzyme solution (145.27 IU/mL; Blend Cellulase SAE 0020, Sigma) was added to each tube. The tubes were incubated at 37 °C in a water bath.

At predetermined time points, samples were withdrawn for visual observation (via images) and glucose quantification; the latter was conducted using the DNS colorimetric method.^44^ Absorbance readings were taken at 540 nm using a spectrophotometer (LKB Biochron–Novaspec II). All tests were performed in triplicate.

#### 2.3.9 Degradation Assay of Biocomposites in an Enzymatic Solution

The enzymatic degradation of biocomposites was performed following the same principles described in Section 2.3.8, but at a single pH (8.5). Visual documentation of the samples was carried out throughout the assay. After 12 h of enzymatic exposure, the samples were centrifuged at 3800 rpm for 10 min. The supernatant was analyzed to reduce sugar content using the DNS assay to monitor the degradation process. The solid residue from centrifugation was subsequently subjected to X-ray diffraction (XRD) analysis to assess structural alterations relative to the undegraded samples.

## 3 Results and Discussion

### 3.1 Scanning Electron Microscopy with Dispersive Energy Spectroscopy (SEM/EDS)

The formation of an apatite layer, commonly referred to as secondary apatite, on the surface of an implant under simulated body conditions (SBF) is widely recognized as a key indicator of in vivo bioactivity, as it reflects the material’s ability to bond with natural bone and provides valuable insight into its bioactive potential under in vitro conditions.^45^ In Figure 2A, a dense layer of HAp crystals, in the form of rosettes and lamellar structures, can be observed over the BNC/HAp biocomposite, indicating the effectiveness of biomimetic mineralization for HAp nucleation. It was observed that the BNC nanofibrils promoted sites for secondary apatite nucleation after immersion in SBF (Figures 2A and C). After the bioactivation test, the composites were analyzed by SEM-EDS, confirming the deposition of Ca- and P-based material on the membrane surface (Figures 2B and S1). Other authors also observed the rosette structure and reported that apatite nucleated at a distinct location and formed outward in a radial pattern.^35,45^

**Figure 2:**
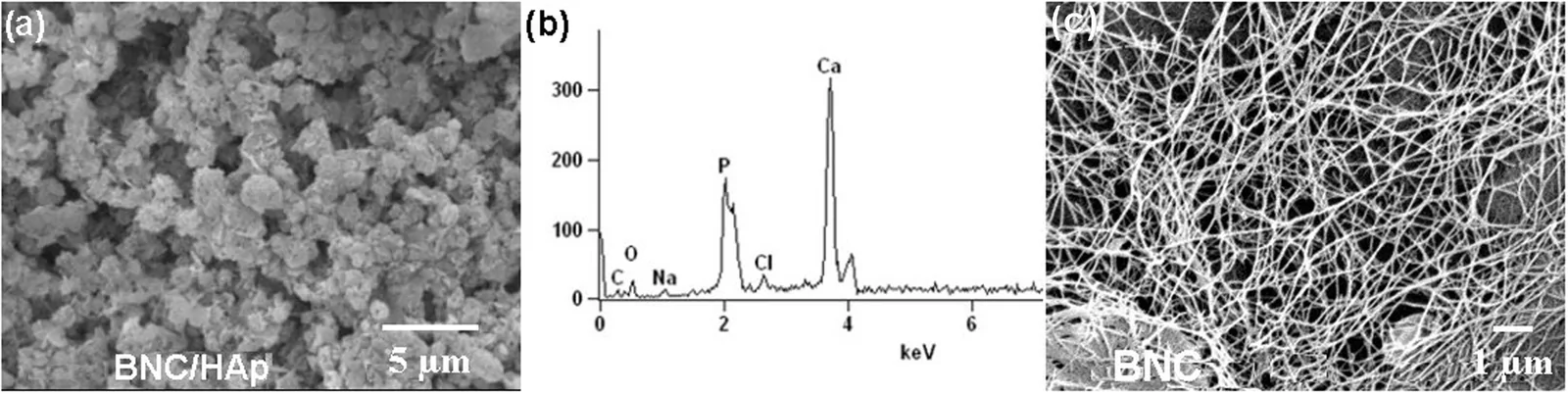
Surface micrographs of the (A) BNC/HAp biocomposite, (B) EDS of the BNC/HAp biocomposite (Ca/*P* = 1.16), and (C) BNC.

Figure 3 shows that substituting calcium with Mg, Cu, Zn, and Sr ions in the biocomposite led to more pronounced changes in density rather than morphology. The emergence of concentric aggregations or clusters suggests multiple nucleation events occurring at specific nanofibril sites. The BNC/MgAp 5% and BNC/SrAp 1% samples (Figures 3C and J) exhibited more lamellar-shaped crystals. Meanwhile, BNC/MgAp 1% and BNC/ZnAp 3% (Figures 3A and H) showed flake-like crystal deposits. In contrast, all BNC/CuAp compositions displayed a distinct behavior, characterized by a dense and continuous coating across the entire BNC surface (Figure 3D–F). This behavior affected the porosity of the biocomposite. This observation aligns with the findings of Huang et al.,^46^ who reported that Cu substitution in HAp increases material density and results in lower porosity than pure HAp coatings. Conversely, Nagyné-Kovács et al.^47^ found no substantial morphological alterations in HAp crystals upon doping with Sr.

**Figure 3:**
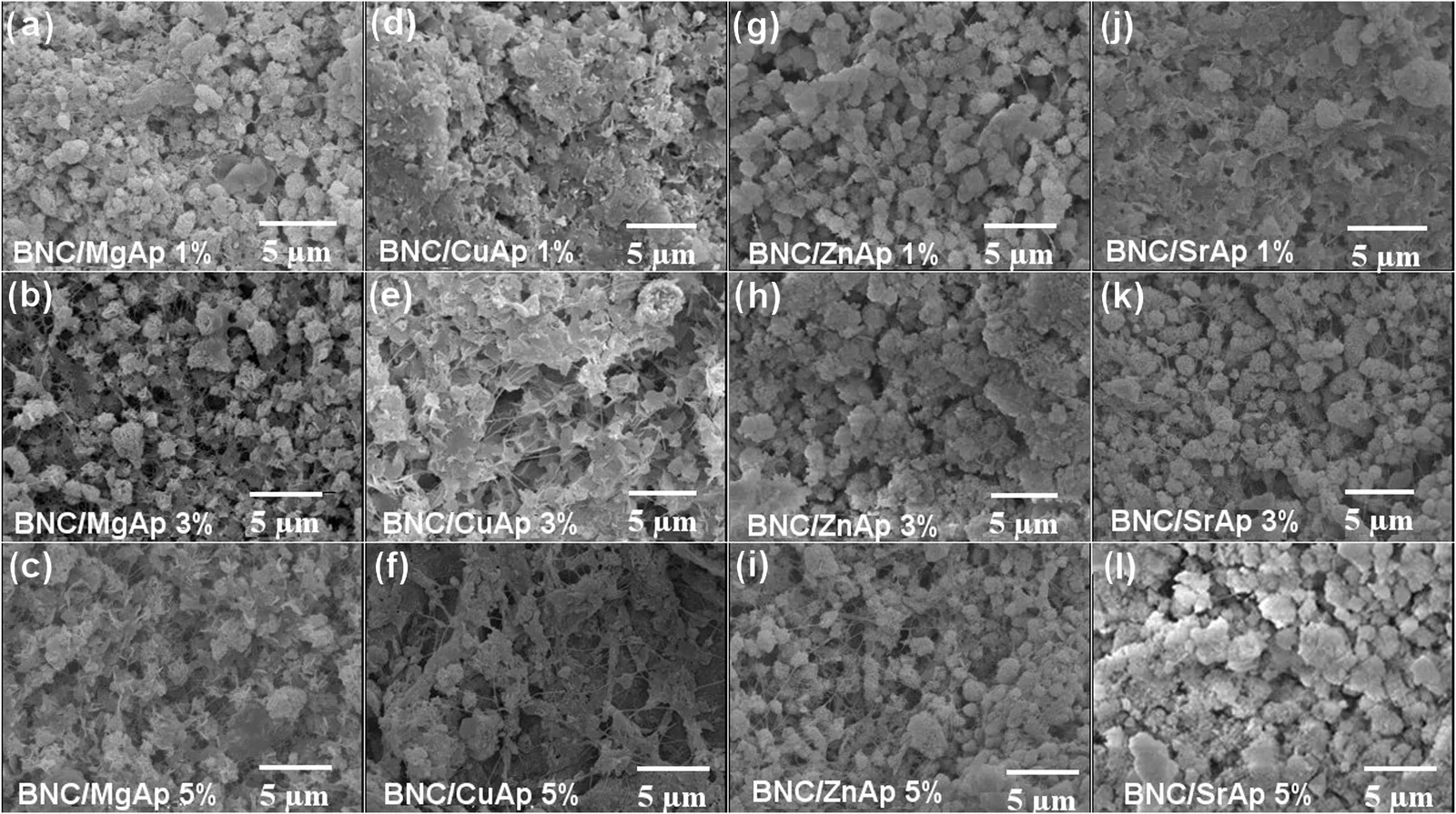
Micrographs of the surface of incorporated biomaterials after 14 days of immersion in SBF: (a–c) BNC/MgAp 1, 3 and 5%; (d–f) BNC/CuAp 1, 3 and 5%; (g–i) BNC/ZnAp 1, 3 and 5%; (j–l) BNC/SrAp 1, 3 and 5%.

EDS analysis detected Ca and P elements in all samples (Figure S1). However, phosphorus detection was affected by peak overlap. Therefore, a reliable Ca/P ratio could not be obtained from the EDS measurements for the incorporated biomaterials. Traces of chlorine, sodium, carbon and oxygen were also observed in the spectrum. The Cl and Na were attributed to residues of the reagents CaCl_2_ and Na_2_HPO_2_ used in the mineralization process. Mg were detected in the BNC/MgAp 3% and 5% samples (0.40 ± 0.04 and 0.66 ± 0.09 wt %, respectively) and Sr in the BNC/SrAp 3% and 5% samples (0.33 ± 0.06 and 0.45 ± 0.08 wt %, respectively). The absence of detectable ions in the remaining samples is likely due to their low concentrations.

### 3.2 Fourier Transform Infrared Spectroscopy Analysis with Attenuated Total Reflectance (FTIR/ATR)

The FTIR spectra of the BNC/HAp biocomposite after 14 days of bioactivation in SBF are shown in Figure 4. All samples exhibited a band in the 3500–3200 cm^–1^ region, which is associated with the hydroxyl groups in cellulose. Furthermore, the slight shift observed in BNC/HAp toward the band attributed to intramolecular hydrogen bonding (∼3500 cm^–1^) confirms the strong interaction between the OH group and apatite. The chemical interaction between HAp and BNC stabilizes the composite, maintaining the mechanical integrity necessary for bone replacement.^48^

**Figure 4:**
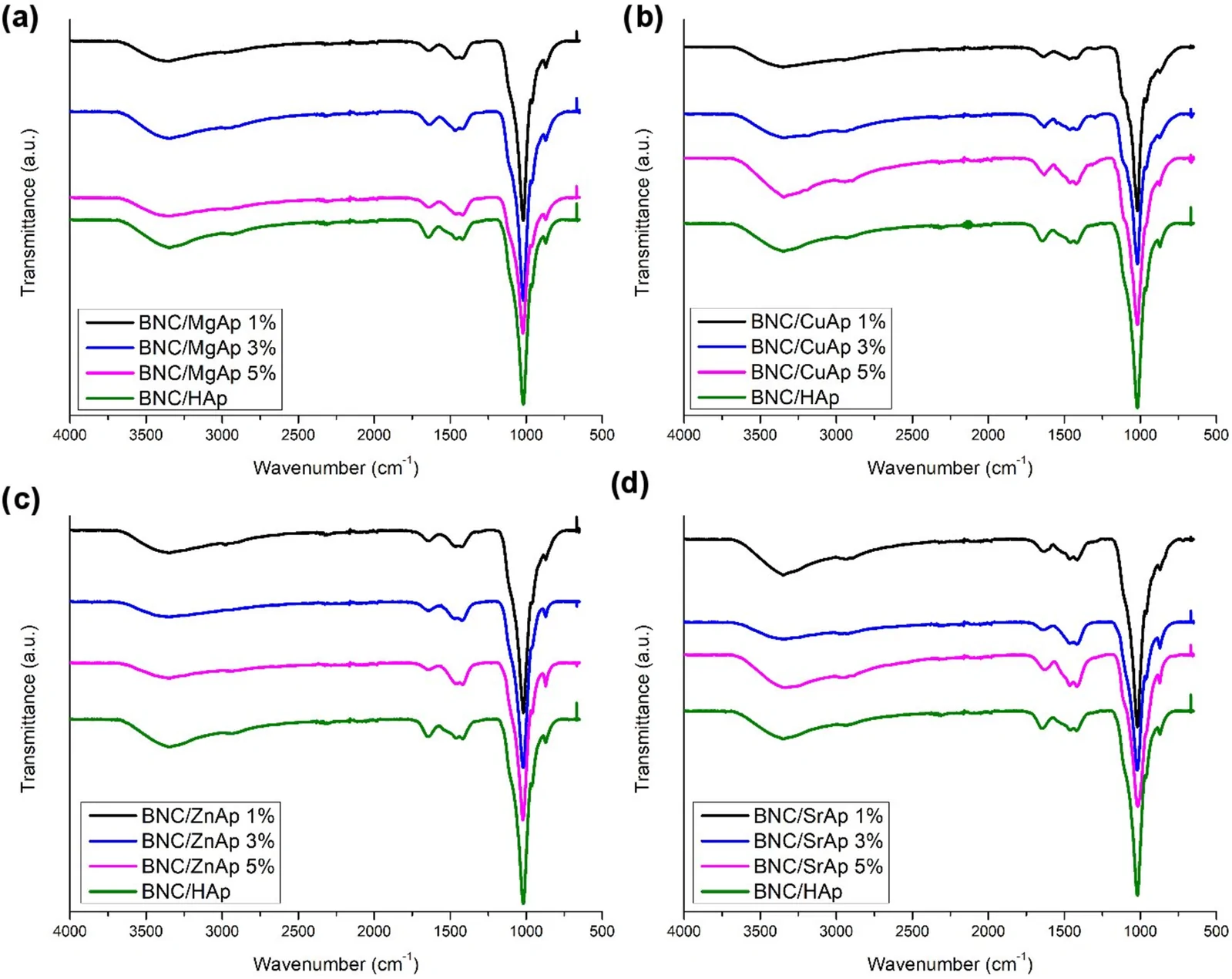
FTIR/ATR spectra of BNC/HAp biocomposites and: (A) BNC/MgAp; (B) BNC/CuAp; (C) BNC/ZnAp, and (D) BNC/SrAp after 14 days of bioactivation in SBF.

Another feature that stands out in the biocomposites is the suppression of the bands at 1160 and 1050 cm^–1^, which correspond to the C–O–C asymmetric elongation modes and C–O bond elongation of BNC, indicating the presence of well-linked chemical groups of BNC and HAp improving the stabilization of the biocomposite.^49^ The bands observed for the BNC and biocomposites in Figure 4 are described in the Supporting Information (Table S1).

In all spectra, the predominance of bands at 1020 and 960 cm^–1^ corresponds to the vibrational modes of the P–O bonds, demonstrating the presence of phosphate groups.^35,50^ However, the 960 cm^–1^ band appeared as a shoulder of the strongest band at 1020 cm^–1^. In addition, the bands at 1418 and 872 cm^– 1^, corresponding to the stretching mode of the CO_3_^2^ ions, suggest the absorption of atmospheric CO_2_. This corroborates the results observed in EDS analysis, which indicated C and O peaks attributable to the presence of CO_3_^2^. Therefore, according to Saska et al.,^48^ their analysis suggests the formation of a carbonate-containing apatite, similar to bone tissue apatite deposited on BNC nanofibers.

Predoi et al.^51^ attributed type B carbonation to the 1461 and 1454 cm^–1^ bands in their MgAp samples. Popa et al.^52^ also observed the presence of the carbonate functional group in their Zn-doped HAp nanoparticles, assigned to the same bands. Durdu et al.^45^ produced steel materials with a coating by deposition of bioactivated hydroxyapatite in SBF. Their samples also showed CO_3_^2^ absorption bands of characteristic elongation mode in slightly substituted carbonated HAp of type A between 1460 and 1465 cm^–1^ and of type B between 1420 and 1425 cm^–1^.

### 3.3 Wide-Angle X-ray Diffractometry (XRD)

Figure 5 presents the XRD patterns of the samples, where two prominent diffraction peaks associated with BNC were detected at 2θ ≈ 14.5° and 22.6°, corresponding to the (100) and (110) planes, respectively.^53^ Characteristic HAp peaks were also identified at 2θ ≈ 26° and 32°, assigned to the (002) and (211) planes. Additional HAp-related reflections were observed at 2θ ≈ 33° (300), 40° (310), 46° (222), and 54° (004), although with lower intensity.^49,53–56^ These results further confirm the incorporation of HAp into the BNC matrix, as previously supported by the FTIR analysis.

**Figure 5:**
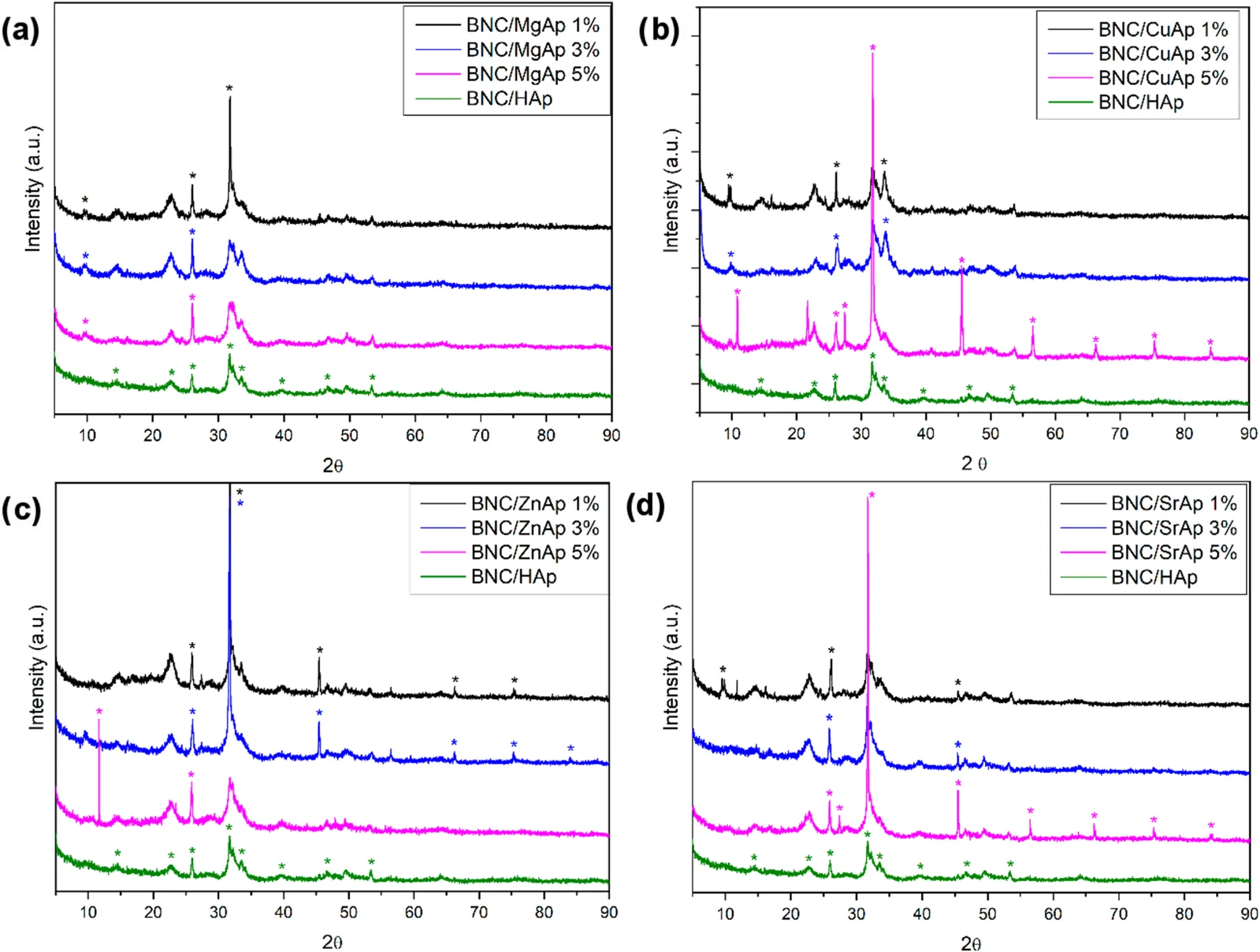
XRD of BNC/HAp biocomposites and: (A) BNC/MgAp; (B) BNC/CuAp; (C) BNC/ZnAp, and (D) BNC/SrAp after 14 days of bioactivation in SBF.

In the modified biocomposites, a noticeable increase in the intensity of peaks at 2θ ≈ 26° and 31.8° was observed compared with the standard BNC/HAp sample, indicating enhanced crystallinity of the material. The gradual crystallization of a hydroxyapatite phase is confirmed by the progressive formation of peaks attributed to hydroxyapatite after 10 and 20 days of immersion in SBF.^57^ In the present study, samples were incubated in SBF for 14 days.

Putrakumar et al.^50^ identified additional peaks at 2θ ≈ 35.57° and 38.67° in Cu-modified hydroxyapatite, corresponding to the (11–1) and (111) planes of copper oxide. These peaks were found to increase in intensity in proportion to higher copper content. However, at concentrations below 5%, such reflections were not prominent in their work. In the current work, subtle reflections at similar positions were detected in the BNC/CuAp samples containing 1% and 3% Cu.

Giannoulatou et al.^57^ also reported new peaks at 2θ ≈ 27.74°, 30.89°, and 34.12° in Cu-doped magnesium–calcium-silicate bioactive glass after 20 days of SBF immersion. These were attributed to the crystalline whitlockite phase (Ca_18_Mg_2_H_2_(PO_4_)_14_), resulting from partial substitution of calcium phosphate by magnesium. In this study, weak peaks around 27.74° and 34.12° were detected, likely due to the low concentration of Mg used.

Additionally, Wang et al.^54^ produced HAp scaffolds doped with Sr and Zn and observed two new peaks at 31.7° and 34.7° in the Zn-doped materials. The 31.7° peak was attributed to a small amount of tricalcium phosphate containing Zn, while the 34.7° peak was attributed to Zn-TCP formation after Zn-Hap. In this work, only a subtle peak at 31.8° was observed in the BNC/ZnAp samples with 1% and 3% Zn.

In the Sr-doped samples, a diffraction peak at 2θ ≈ 31.8° was observed, becoming progressively sharper and more intense as the Sr content increased. This behavior suggests improved crystallinity with higher Sr incorporation. Similar findings were reported by Nagyné-Kovács et al.,^47^ who synthesized Sr-doped hydroxyapatite using various Sr/(Ca+Sr) molar ratios. They noted that although no additional peaks associated with strontium were detected, a slight shift in the main diffraction peak toward lower angles was observed, specifically from 2θ ≈ 31.848° to 2θ ≈ 31.705°. This subtle shift was attributed to the substitution of calcium by strontium ions within the HAp crystal lattice.

### 3.4 Thermogravimetric Analysis (TGA)

The thermal degradation behavior of BNC/HAp and the doped biocomposites revealed six distinct mass loss stages, as depicted in Figure 6. There was an initial mass loss around 100 °C, referring to dehydration of the cellulose and a second stage that may result from the vaporization of hydroxyapatite crystallization water at around 120 °C.^48^ A very mild stage at around 220 °C was observed in almost all samples, with mass losses ranging from 2.3% to 9.73%. Cox et al.^58^ prepared and characterized a nanophase of Sr-, Mg-, and Zn-substituted hydroxyapatite via aqueous precipitation and highlighted that this may be associated with gradual later water loss. The fourth and most striking thermal event occurs at T_peak4_ around 320 to 330 °C with a pronounced mass loss, 17% to 28%. These refer to the cellulose degradation process, including depolymerization, dehydration, and decomposition of glycosyl units, followed by the formation of a carbonized residue.^48,59^

**Figure 6:**
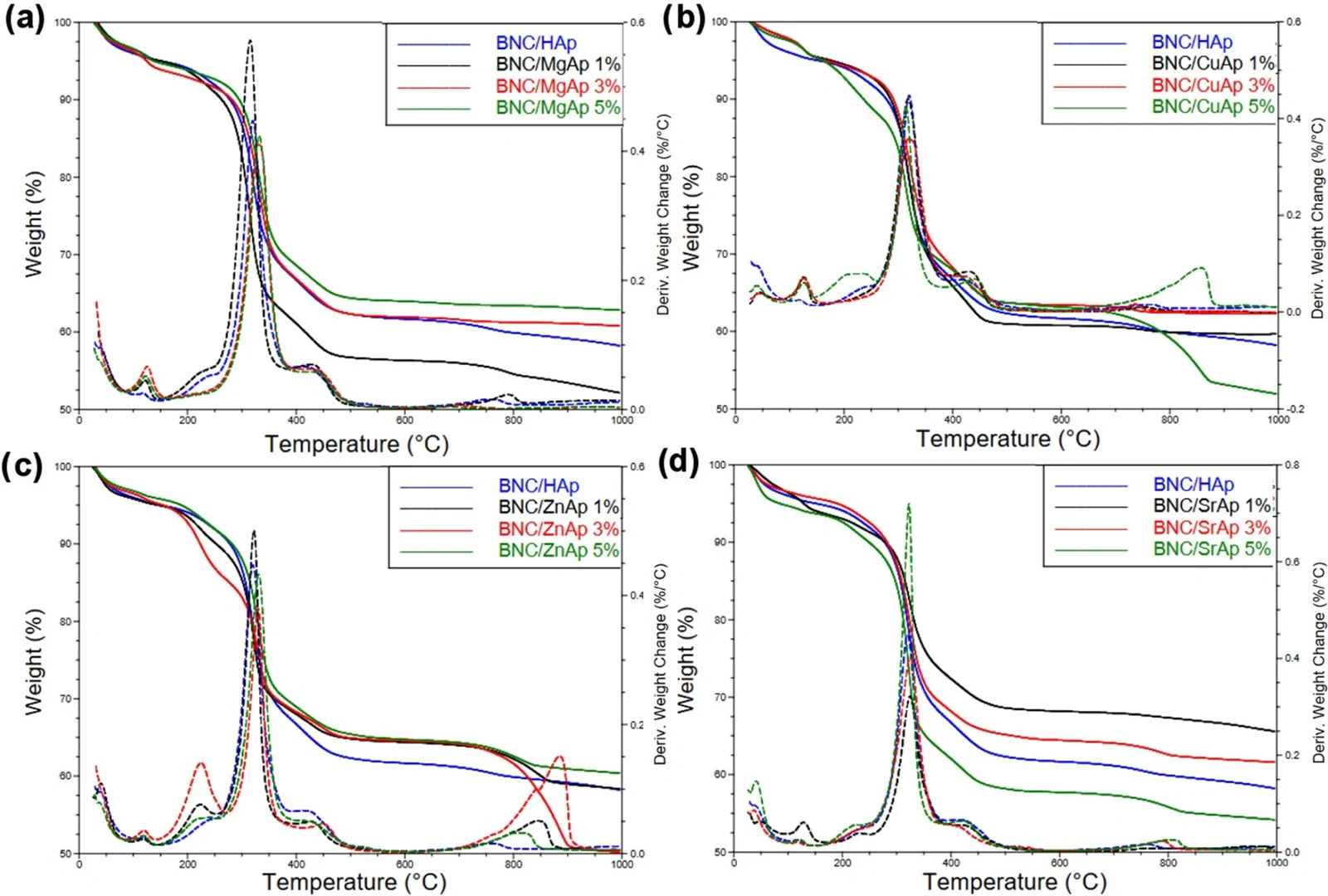
TG and DTG curves of BNC/HAp biocomposites (standard) and: (A) BNC/MgAp; (B) BNC/CuAp; (C) BNC/ZnAp, and (D) BNC/SrAp after 14 days of bioactivation in SBF.

The fifth stage, around (T_peak5_) 412 to 452 °C, is due to the degradation of carbonaceous residues from the BNC.^60,61^ The final stage (T_peak6_), occurring between 702 and 856 °C, was associated with the decomposition of carbonated apatite, which, according to Zhang et al.^62^ is related to the mass loss of carbonated apatite. This result also agrees with FTIR data, which showed the decomposition of nonstoichiometric apatites into whitlockite; this event occurs above 600 °C.^58^ These data are in the Supporting Information (Table S2).

Miyaji et al.^63^ reported in their work that the mass losses that occur between 750 and 850 °C were related to the decomposition of apatite to form tricalcium phosphate (TCP) (Ca_3_(PO_4_)_2_), and this loss increases with the increasing percentage of Zn, that is, Zn can promote the transformation of apatite into TCP. The highest values in this temperature range were also observed in this study for Zn-containing biocomposites. In general, some biocomposites with different metallic ions also showed this effect, raising the T_peak6_ in the biocomposite.

All samples exhibited a high residual mass after heating, ranging from 50.16% to 65.6%, indicating that approximately half of the biocomposite consists of deposited HAp.^48^ Regarding the tested biocomposites, at T_peak4_, associated with the main cellulose degradation stage, the incorporation of metallic ions shifted the maximum degradation temperature to higher values for all compositions except BNC/MgAp 1% and the BNC/CuAp samples, compared with the BNC/HAp control (320 °C). This shift suggests a slight improvement in the thermal stability of the main degradation stage. However, the corresponding mass loss did not follow the same behavior. Mg-doped biocomposites exhibited relatively high mass losses despite the increase in T_peak4_, whereas Zn-containing samples, particularly BNC/ZnAp 3%, showed both higher degradation temperatures and lower mass losses. In contrast, Cu incorporation generally shifted T_peak4_ to lower temperatures, although the associated mass loss varied with concentration, with BNC/CuAp 5% exhibiting the lowest value among the Cu-doped samples. The slight increase in the maximum degradation temperature observed for the BNC/MgAp (3% and 5%), BNC/ZnAp, and BNC/SrAp samples indicates that incorporating bioactive ions did not compromise the thermal stability of the BNC/HAp matrix.

### 3.5 Cytotoxicity Assay

The cytotoxicity assessment via the MTS assay is presented in Figure 7. It is important to note that lower cell viability percentages indicate a higher cytotoxic potential of the tested materials. According to ISO 10993–5:2009, materials are considered noncytotoxic when cell viability exceeds 70% relative to the control.

**Figure 7:**
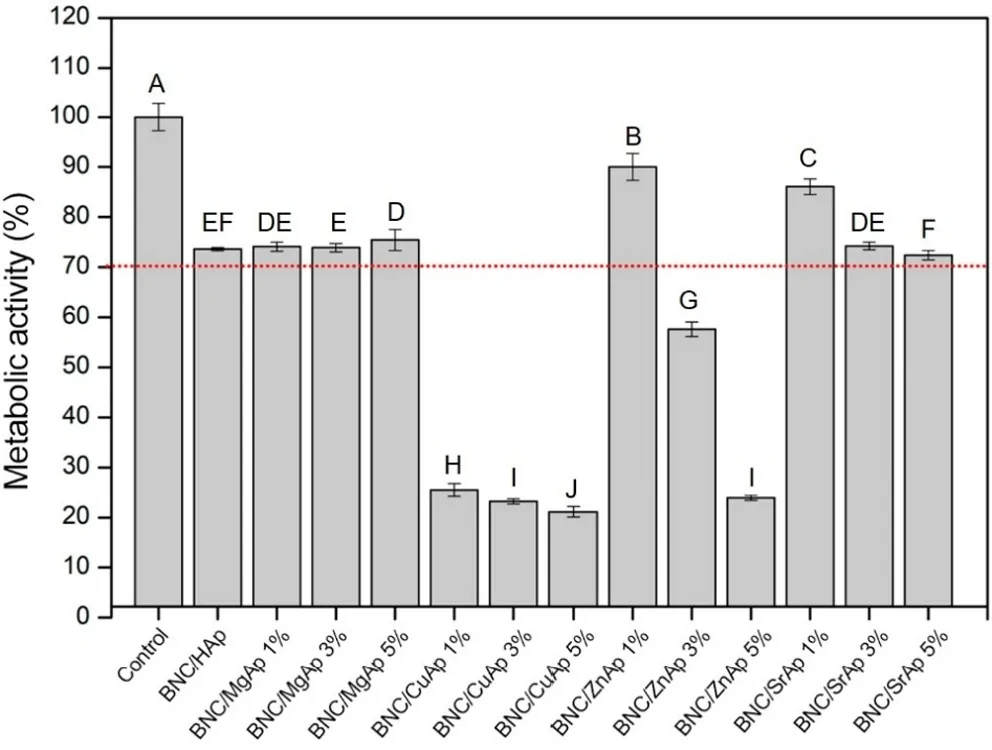
Result of cell viability analysis for BNC/HAp, BNC/MgAp, BNC/CuAp, BNC/ZnAp and BNC/SrAp biocomposites after 14 days of bioactivation in SBF (*n* = 9 ± s.d.; different letters indicate statistically significant differences between groups according to one-way ANOVA followed by Tukey’s post hoc test (α = 0.05)).

The results demonstrated that all Mg-containing biocomposites exhibited no important difference in cell viability compared to the BNC/HAp control, which is favorable for biomedical applications. In contrast, all Cu-doped samples showed considerable cytotoxicity, reducing cellular metabolic activity to approximately 20%. Among the Zn-doped samples, BNC/ZnAp 1% showed the most promising outcome, maintaining about 90% cell viability. However, increasing the Zn concentration led to a marked decline in cell metabolic activity, suggesting a concentration-dependent cytotoxic response. Notably, Sr-doped composites exhibited good biocompatibility. The BNC/SrAp 1% sample retained nearly 90% of cell metabolic activity, and even at higher concentrations (3% and 5%), the activity remained above 70%.

Favi et al.^64^ investigated the viability of mesenchymal stem cells (hMSCs) cultured on BNC and BNC/HAp biocomposites. The results showed that cells remained metabolically active after 48 h, confirming the materials’ cytocompatibility. Similarly, Kazimierczak et al.^65^ studied scaffolds composed of chitosan/agarose combined with HAp and doped variants: chi/aga/MgAp, chi/aga/ZnAp, and chi/aga/Mg-ZnAp. Each formulation included 30% HAp or metal ions, either singly or as a combination (15% each of Mg and Zn). After 24 h of exposure, scaffolds containing Mg^2+^ ions demonstrated enhanced metabolic activity in MC3T3-E1 osteoblastic cells, which the authors attributed to Mg^2+^’s ability to promote osteoblast viability through improved intercellular communication.

In contrast, Chi et al.^66^ synthesized Cu-doped apatite (CuAp) microspheres via ion exchange and pneumatic molding and assessed cytotoxicity using bone marrow-derived mesenchymal stem cells (BMMSCs). Their findings indicated a significant decrease in cell viability during the initial 24 to 72 h, with cytotoxicity directly proportional to the Cu^2+^ concentration, suggesting that copper ions exhibit notable cytotoxic effects, particularly in early exposure stages.

Popa et al.^52^ evaluated ZnAp nanoparticles for cytotoxicity toward HepG2 cells and found a moderate effect on cell viability (around 20%). They attributed this result to the reduction in particle size due to increased Zn content. Zinc ions are known to induce cytotoxicity at high concentrations, with cytotoxic effects increasing in a concentration-dependent manner.^67,68^ In the present study, this behavior was clearly observed, as BNC/ZnAp 1% maintained high cellular metabolic activity, whereas increasing the Zn concentration significantly reduced cell viability.

Nguyen et al.^69^ studied Sr-doped calcium phosphate coatings on titanium substrates for their biocompatibility, using MC3T3-E1 osteoblasts. After 7 days, the coatings with the highest Sr content (2.13%) exhibited superior cell viability compared to the control. The study suggested that Sr ions may act as chemical stimuli, enhancing cell adhesion and proliferation, which are crucial for bone tissue regeneration and implant integration.

Based on the results for all biocomposites produced and considering the best performance on metabolic activity, only the compositions BNC/MgAp 1%, BNC/ZnAp 1% and BNC/SrAp 1% were selected for subsequent assays.

### 3.6 Cell Viability and Proliferation Assay

Figure 8 presents data on the metabolic activity of osteoblasts cultured on the BNC/HAp, BNC/MgAp 1%, BNC/ZnAp 1%, and BNC/SrAp 1% biocomposites over 1, 7, 14, and 21 days.

**Figure 8:**
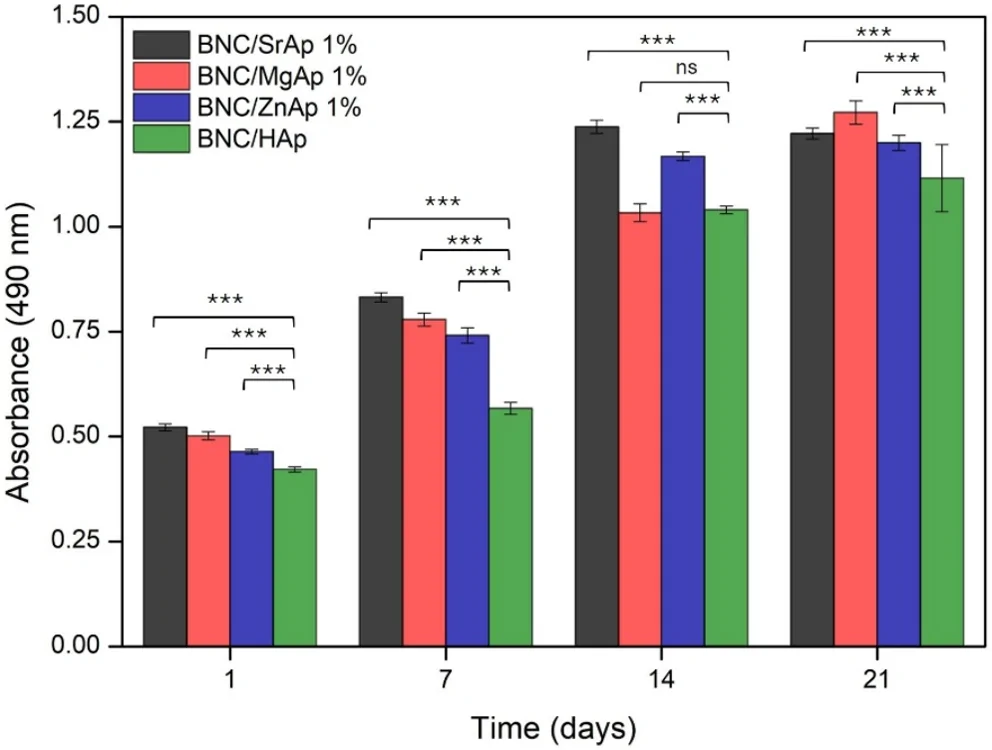
Cell viability assay with osteoblasts MC3T3 on the biocomposites BNC/HAp, BNC/MgAp 1%, BNC/ZnAp 1% and BNC/SrAp 1% in the periods of 1, 7, 14, and 21 days (*n* = 8 ± s.d.; Statistical significance was determined using the Mann–Whitney *U* test by comparing each ion-substituted biocomposite with the BNC/HAp control at the corresponding time point. *** *p* < 0.001; ns, not significant).

A notable increase in cell proliferation was observed after 7 days in all biocomposites compared to the control group, suggesting favorable early stage cell adhesion. This effect may be attributed to the strong interaction between the cells and the biomaterials, promoting early adhesion.^38^

Over time, cellular responses diverged depending on the material, suggesting that each ion substitution contributes differently to the osteoblastic response throughout the culture period. At 14 days, the BNC/SrAp 1% and BNC/ZnAp 1% samples supported higher cell proliferation than the control and other biocomposites, indicating enhanced compatibility at this stage. The early enhancement promoted by BNC/SrAp 1% may be associated with the incorporation of Sr^2+^ into HAp, which has been reported to stimulate osteoblast proliferation and early osteogenic differentiation, enhance bioactivity and cell adhesion, and reduce osteoblast apoptosis, thereby promoting a favorable microenvironment for bone regeneration.^70,71^ Likewise, zinc, when combined with HAp, enhances bioactivity by improving osteoblast viability and promoting osteogenic activity.^68^ Zinc enhances bone formation and mineralization by stimulating collagen production and increasing alkaline phosphatase (ALP) activity, a marker of osteoblast differentiation.^71^ By day 21, the metabolic activity of osteoblasts on BNC/SrAp 1% and BNC/ZnAp 1% remained consistent with the 14-day results. However, BNC/MgAp 1% stood out, showing a significant increase in cell proliferation compared to the control and the other samples, and was the only biocomposite to demonstrate a continuous increase throughout the entire 21-day period. This consistent growth observed for BNC/MgAp 1% may be linked to the effects of magnesium on cellular behavior, including adhesion, migration, proliferation, and differentiation.^68^

This positive influence on the cell growth of the biocomposites compared to the standard can also be seen in the analysis of the distribution of the medians considering the entire assay for the BNC/HAp standard (Figure 9). The Mann–Whitney *U* test revealed significant differences (*p* < 0.05) between each ion-substituted biocomposite and the BNC/HAp control at all time points, except for BNC/MgAp 1% at day 14 (*p* = 0.5737), for which no statistically significant difference was observed. This result is still considered positive due to the general behavior over time and because, in this case, it is on an equal footing with the control.

**Figure 9:**
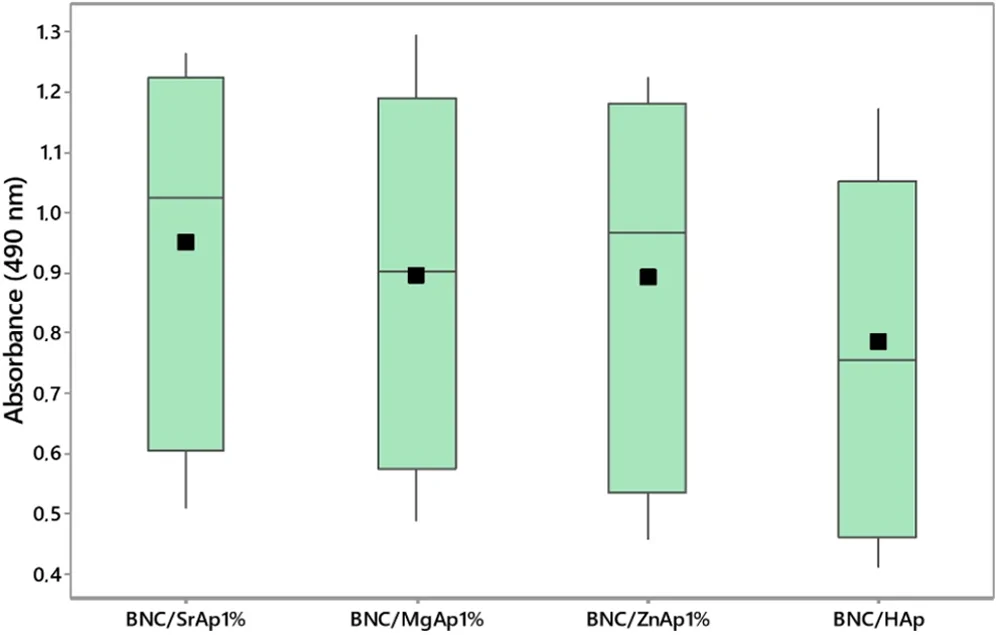
Distribution of medians of cell growth data referring to cell adhesion assay with osteoblasts MC3T3 on the biocomposites BNC/HAp, BNC/MgAp 1%, BNC/ZnAp 1% and BNC/SrAp 1%.

These findings demonstrate that the biocomposites promoted higher cellular viability than the control across different time points, confirming their good cytocompatibility. These results are very important because cell viability is essential for tissue regeneration and the entire trauma repair process.^40^

### 3.7 Preliminary Study of BNC Degradation in Enzyme Solution

Cellulase is an enzyme produced mainly from fungi such as *Trichoderma sp*, *Aspergillus sp* and *Penicillium sp*., therefore, its optimal activity is usually close to pH 5.^72^ However, considering the optimal pH for alkaline phosphatase activity between 8.0 and 10.5,^73^ which, in this work, is being considered as an indicator of bone formation, and the physiological pH of the body around 7.4 ^34^ a preliminary assay was conducted to verify the degradation of pure BNC using a cellulase enzymatic solution to verify the influence of these pHs on the cellulase enzymatic activity.

Figure 10 shows the visual outcomes of BNC membranes subjected to enzymatic degradation in phosphate buffer solutions at pH 7.5 and 8.5. At pH 7.5, signs of membrane fragmentation were observed as early as 2 h into the assay. After 6 h, only small fragments in suspension were visible, which persisted throughout the 48-h testing period. In contrast, membranes incubated at pH 8.5 remained intact at 2 h, exhibited minor cracks by 6 h, and showed significant fragmentation only after 12 h. Complete disintegration into suspended fragments was observed at 24 h, with the fragmented state maintained until the end of the assay at 48 h. The enzymatic degradation of BNC by cellulases involves the fibrillation of cellulose fibers into finer cellulose microfibril (CMF) bundles. This process initiates via longitudinal cracking of the BNC network, after which CMF hydrolysis accelerates, while some more compact fibril bundles can retain their structural integrity, even under extended enzymatic exposure.^74^

**Figure 10:**
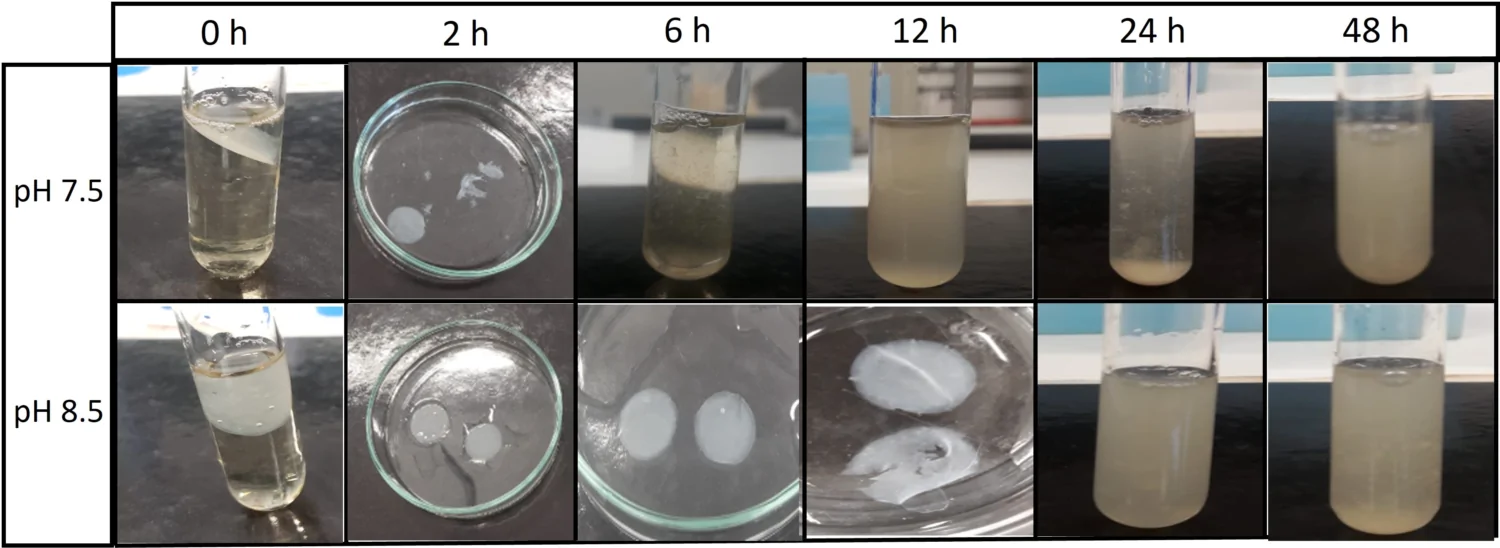
Visual analysis of the enzymatic biodegradation process of BNC at pH 7.5 and 8.5.

Figure 11 presents data on the concentration of reducing sugars as a function of time, as a product of BNC degradation by cellulases. The concentration of reducing sugar increased during the first 6 h, suggesting rapid initial degradation. After 12 h, however, the glucose levels plateaued at both pH values, indicating a decline in enzymatic efficiency. Interestingly, this result contrasts slightly with the visual observations (Figure 10), in which fragmentation appeared to progress beyond the 6-h mark, particularly at pH 8.5. This discrepancy suggests that while macroscopic degradation continued, the enzymatic release of reducing sugars reached a saturation point early in the process.

**Figure 11:**
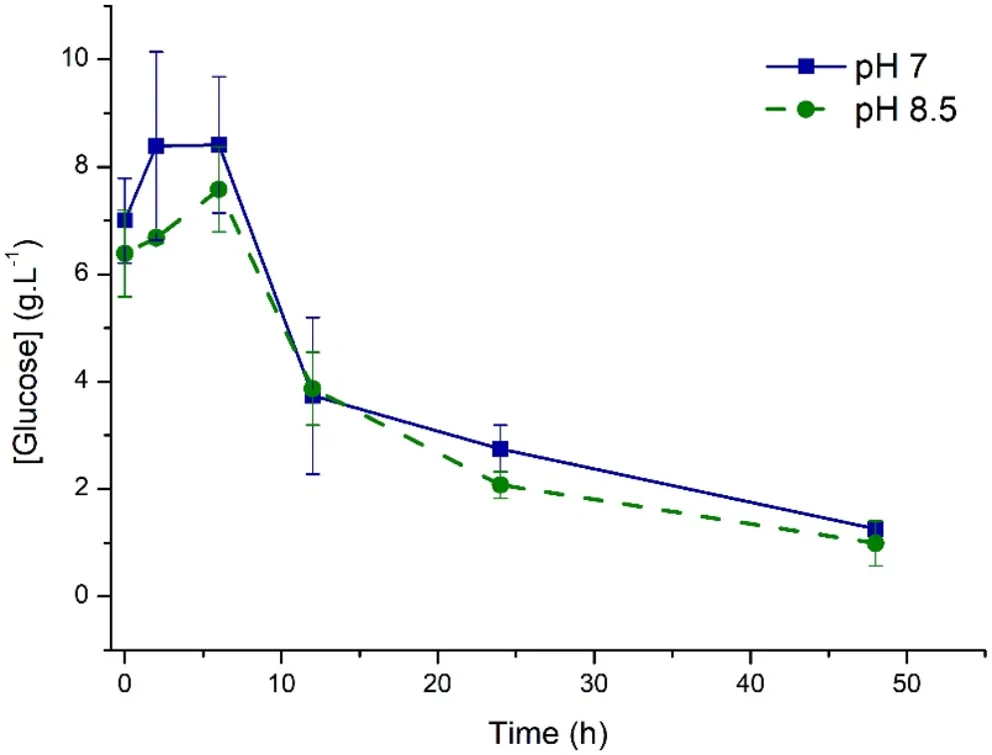
BNC biodegradation assay compared at pH 7.5 and 8.5, analysis of sugar production as a function of the enzymatic attack (*n* = 3 ± s.d.).

Another particularly interesting point is the decrease in glucose concentration over time, even though no volume was removed from the reaction medium. Penttilä et al.^74^ analyzed the enzymatic hydrolysis of bacterial cellulose-hemicellulose composites using a commercial cellulase mixture (Celluclast) and also reported a reduction in the *shoulder* characteristic of the graph after 10 h of testing. Two propositions can be made regarding this behavior: (1) the enzyme blend likely contains endoglucanases, exoglucanases, and β-glucosidase, whose synergistic activity promotes efficient BNC breakdown at first, but may later be inhibited by the accumulation of glucose itself;^75^ and (2) as also noted by Penttilä et al.,^74^ BNC degradation occurs through fibrillation into more resistant cellulose microfibril bundles, as already cited in this present work, which reduces enzyme accessibility and slows the hydrolysis process in later stages.

Although biodegradation was generally more intense during the first 6 h at pH 7.5, the standard deviations indicate that both pH 7.5 and 8.5 produced comparable behavior overall. Based on these findings and considering that alkaline phosphatase, used here as an indicator of bone formation, has optimal activity between pH 8.0 and 10.5, the subsequent biocomposite degradation assays were conducted exclusively at pH 8.5. This decision also aligns with the physiological relevance of this pH range for bone regeneration.

### 3.8 Degradation Assay of Biocomposites in an Enzymatic Solution

The enzymatic biodegradation behavior of the biocomposites showed only slight variations relative to pure BNC. However, the degradation process appeared to occur more slowly in the early stages. This delay may be attributed to the apatite coating on the BNC microfibrils, which could act as a physical barrier, limiting initial enzyme access.

Figure 12 shows representative photographs of BNC/HAp, BNC/MgAp 1%, BNC/ZnAp 1%, and BNC/SrAp 1% samples subjected to enzymatic degradation in phosphate buffer at pH 8.5. The degradation pattern resembled that observed for pure BNC at the same pH. The first visible signs of structural weakening appeared only after 12 h of exposure. Interestingly, after 24 h, the BNC/ZnAp 1% and BNC/SrAp 1% samples still appeared structurally intact. However, by the end of the assay (48 h), all samples were similarly fragmented.

**Figure 12:**
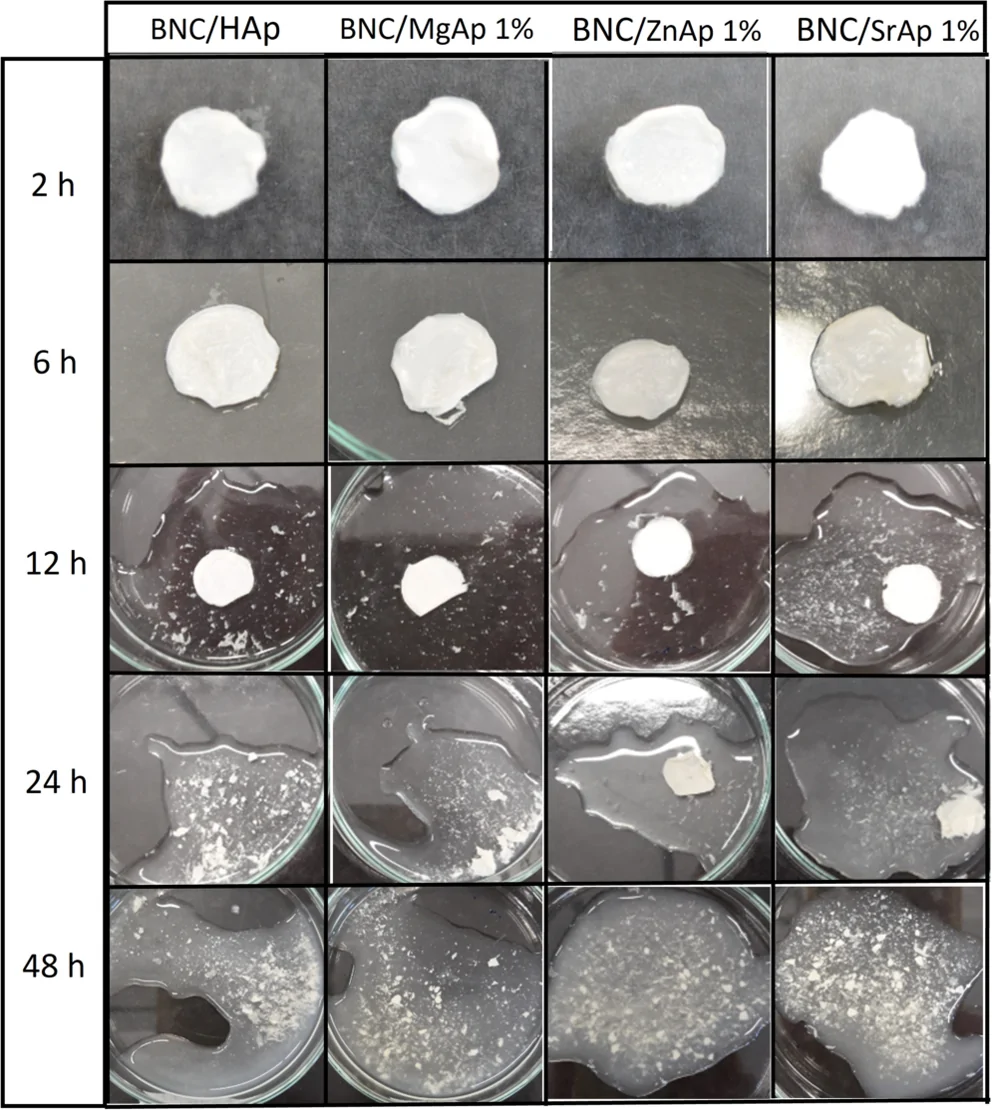
Visual analysis of BNC/HAp, BNC/MgAp 1%, BNC/ZnAp 1% and BNC/SrAp 1% biocomposites subjected to enzymatic degradation in phosphate buffer pH 8.5 as a function of time.

Figure 13 presents the reducing sugar concentration over time during enzymatic degradation at pH 8.5. In the magnified section of the graph, it is possible to observe that BNC/MgAp 1% and BNC/HAp exhibited higher sugar release within the first 2 h. After 6 h, the BNC/MgAp 1% sample continued to show elevated sugar levels, while BNC/HAp levels began to decline. The BNC/SrAp 1% sample reached its peak sugar release at 6 h, whereas the BNC/ZnAp 1% sample showed minimal variation throughout the assay. These results agree with the visual analysis.

**Figure 13:**
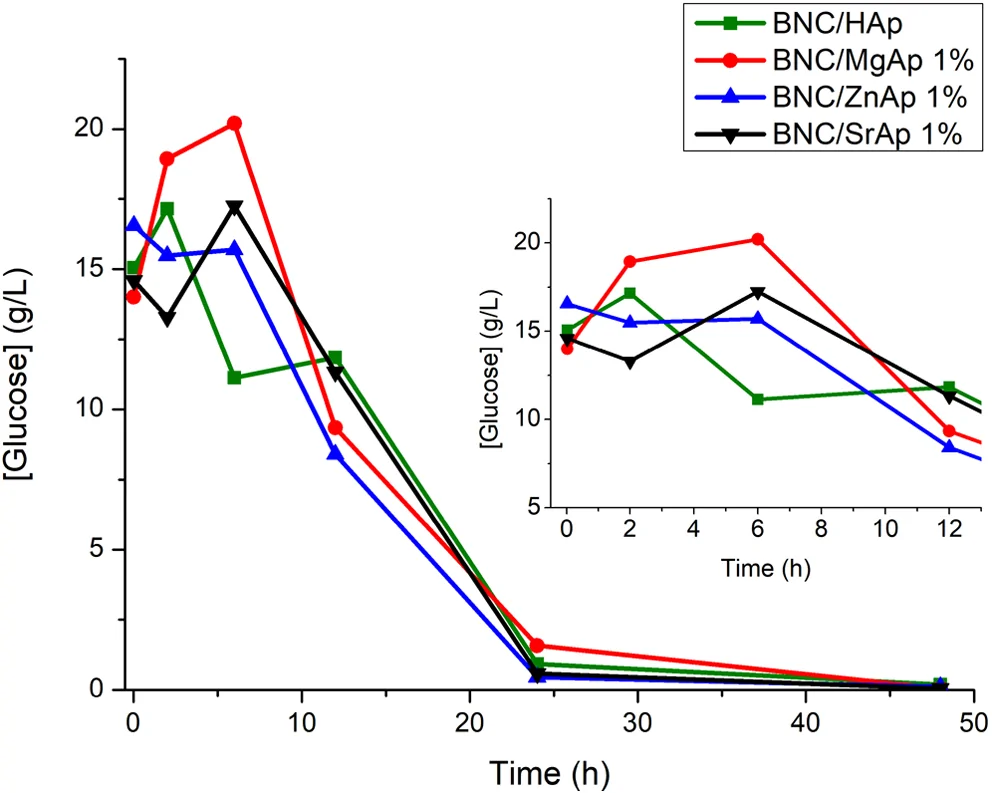
Analysis of sugar production as a function of enzymatic attack on BNC/HAp, BNC/MgAp, BNC/ZnAp and BNC/SrAp biocomposites in pH 8.5 phosphate buffer as a function of time.

In the case of the biocomposites, unlike the pure BNC degradation assay, the variable introduced is the incorporation of partially substituted apatites containing Mg^2+^, Zn^2+^, and Sr^2+^ ions, in addition to Ca^2+^ as the standard component. When released into an aqueous environment over time, these ions may exert an inhibitory effect on enzyme activity. Costa (2013),^76^ investigated this phenomenon by isolating two β-glycosidases from the fungus *Penicillium chrysogenum*. In addition to various enzymatic characterizations, the study assessed the influence of different metal ions on the enzymes’ hydrolytic performance. The results showed that Fe^3+^, Pb^+^, and Ba^2+^ had the most pronounced inhibitory effects. Lesser but still notable inhibition was observed with Cu^2+^ > Hg^+^ > Mn^2+^ > Co^2+^ > Ca^2+^ > Zn^2+^. Mg^2+^ was found to inhibit only one of the two enzymes studied, with an effect comparable to that of Zn^2+^. According to the author, these ions may interfere with the enzyme’s thiol groups, which are crucial to the catalytic center and to maintaining the protein’s tertiary structure. This interference can lead to enzyme inhibition. Therefore, the presence of these ions in the biocomposites tested in the present study may help explain the differences observed in enzymatic degradation behavior among the various compositions.

### 3.9 Wide-Angle X-ray Diffractometry (XRD)

Figure 14 shows the XRD patterns of the biocomposite samples before and after 12 h of *in vitro* enzymatic degradation. The peaks highlighted by a rectangle (2θ ≈ 14.5° and 22.6°) are attributed to BNC, as previously reported.^51,77^ The peaks indicated by black arrows, located at 2θ ≈ 26°, 32°, 33°, 40°, 46°, and 54°, are characteristic of HAp.^54,55^ All these peaks are clearly observed in the nondegraded samples.

**Figure 14:**
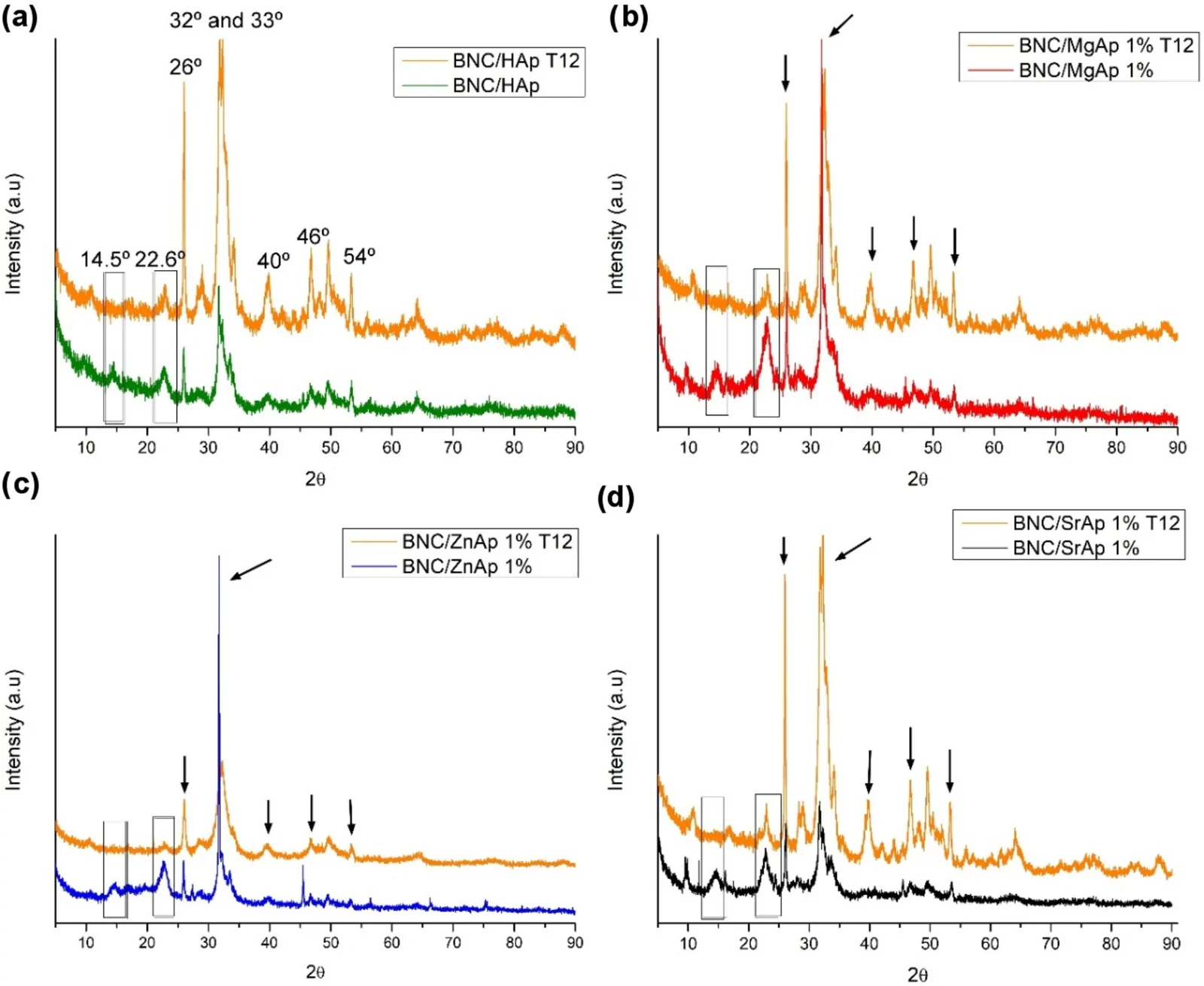
X-ray diffractograms of biocomposites after enzymatic biodegradation. Comparison of samples before and after (T12) the degradation process: (A) BNC/HAp­(standard); (B) BNC/MgAp 1%; (C) BNC/ZnAp 1%, and (D) BNC/SrAp 1%.

Following biodegradation, the intensity of the BNC-related peaks decreased to varying degrees depending on the biocomposite. In the BNC/HAp sample, a strong reduction was observed at 2θ ≈ 14.5°, while the attenuation at 2θ ≈ 22.6° was less pronounced. For the BNC/MgAp 1%, BNC/ZnAp 1%, and BNC/SrAp 1% composites, the peak at 2θ ≈ 14.5° was no longer visible. However, the behavior of the 2θ ≈ 22.6° peak varied across these samples: the most substantial reduction was observed for BNC/ZnAp 1%, followed by BNC/MgAp 1%, while BNC/SrAp 1% showed only a slight attenuation, similar to that observed for BNC/HAp. These findings align with the sugar release data in Figure 13, which shows that sugar production stabilized after 12 h.

Wang et al.^22^ investigated the in vitro degradation of BNC containing varying concentrations of cellulase in FBS at pH 7.4 over 24 weeks. A reduction in the intensity of BNC-specific peaks was observed immediately following enzyme exposure. Nonetheless, even after 10 weeks, the peak at 2θ ≈ 22.6° remained faintly detectable. The authors attributed this to the random cleavage of cellulose chains by endo-1,4-β-d-glucanase, progressively converting crystalline regions into amorphous ones, thereby reducing both crystallinity and peak intensity.

Similarly, Calvini et al.^78^ studied cellulose degradation via oxidation using sodium metaperiodate over a 10-day period. They reported a more substantial reduction at 2θ ≈ 14.5° than at 2θ ≈ 22.6°, suggesting that crystalline regions are more readily oxidized by metaperiodate.

Importantly, the HAp-related peaks remained visible and even more pronounced after enzymatic exposure. Luz et al.^79^ reported similar results in their study of the in vitro degradation of a BNC/HAp composite treated with oxidized BNC (KCl–HCl, pH 1) for 12 h. They observed a decline in BNC peak intensity while the HAp peaks were retained, indicating that HAp had become the dominant phase in the composite. Thus, based on the results presented in this study, it can be concluded that after enzymatic degradation, HAp and the substituted apatites became the predominant components of the material.

As a next step, future work will focus on evaluating enzymatic degradation using different enzyme concentrations, exploring enzyme encapsulation, and conducting degradation studies in a fed-batch system with periodic sampling and enzyme replenishment. These approaches aim to better simulate physiological conditions and enzyme behavior in vivo.

## 4 Conclusions

The presence of phosphate sources within the biocomposites was confirmed through SEM/EDS, FTIR/ATR, XRD, and TGA analyses. At the concentrations tested, the inclusion of Cu demonstrated cytotoxic effects. In contrast, the BNC/MgAp and BNC/SrAp biocomposites at all tested concentrations, as well as BNC/ZnAp at 1%, exhibited minimal adverse effects on cell metabolic activity.

Cationic substitution in the biocomposites enhanced their biological performance compared to the control BNC/HAp, as evidenced by significantly improved osteoblast proliferation. The cellulase enzymes employed in this study were effective at initiating hydrolytic degradation, even under alkaline conditions (pH 8.5), a value selected because it is associated with alkaline phosphatase activity and is relevant to the physiological environment during bone regeneration.

Enzymatic hydrolysis also occurred in the biocomposites, showing only a slight delay compared to pure BNC, likely due to the apatite coating on the nanofibrils, which temporarily hindered enzyme access. Nonetheless, the hydrolysis of the cellulosic matrix and the formation of reducing sugars occurred most prominently within the first 12 h. Fragmentation continued significantly at 24 and 48 h. These observations were supported by XRD analysis, which revealed a marked reduction in the intensity of BNC peaks and a corresponding increase in the HAp peaks, indicating that apatite became the predominant phase in the composite after enzymatic biodegradation.

Finally, adding cellulases to make the biocomposite bioabsorbable proved very promising. Although more studies are still needed in this regard, the results obtained thus far present a strong foundation for future research into the development of biomaterials for bone regeneration applications.

## Supplementary Material



## Data Availability

The data used to support the findings of this study are available from the corresponding author upon request

## Notes

Biomaterials EDS: BNC/MgAp; BNC/CuAp; BNC/ZnAp and BNC/SrAp (Figure S1); data obtained from FTIR analysis for BNC membranes and biocomposites BNC/HAp (standard), BNC/MgAp, BNC/CuAp, BNC/ZnAp, and BNC/SrAp (Table S1); maximum degradation temperature (*T*_peak_) and percentage of mass loss (%) data obtained from the TG and DTG curves of BNC/HAp biocomposites (standard) and BNC/MgAp, BNC/CuAp, BNC/ZnAp, BNC/SrAp (Table S2) (PDF)
