# Supplementary material for: Synthesis and Cytocompatibility of Bacterial Nanocellulose–Hydroxyapatite Biocomposites with Mg, Cu, Zn, and Sr Substitution
Source: ACS Omega. 2026 Sep 21;11(39):59131–46. doi: 10.1021/acsomega.6c04779 (PMC13647905; doi:10.1021/acsomega.6c04779)
Supplement: Supplementary file 1 [file ao6c04779_si_001.pdf]

Synthesis and Cytocompatibility of Bacterial Nanocellulose–Hydroxyapatite Biocomposites with Mg, Cu, Zn, and Sr Substitution

Michele C. Formolo [1,2,5], *Bruna Segat* [1], *Sandro R. K. Junior* [2,3*], *Karina Cesca* [4], *Andrea L. dos S. Schneider* [1,3], *Ana P. T. Pezzin* [3] *and Luismar M. Porto* [5]

[1] Department of Chemical Engineering, University of the Joinville Region, Joinville, 89219-710, Brazil

[2] Postgraduate Program in Health and Environment, University of the Joinville Region, Joinville, 89219-710, Brazil

[3] Postgraduate Program in Process Engineering, University of the Joinville Region, Joinville, 89219-710, Brazil

[4] Program in Food Engineering, Federal University of Santa Catarina, Florianópolis, 88040-900, Brazil

[5] Postgraduate Program in Chemical Engineering, Federal University of Santa Catarina, Florianópolis, 88040-900, Brazil.

*Correspondence: s.sandro@univille.br

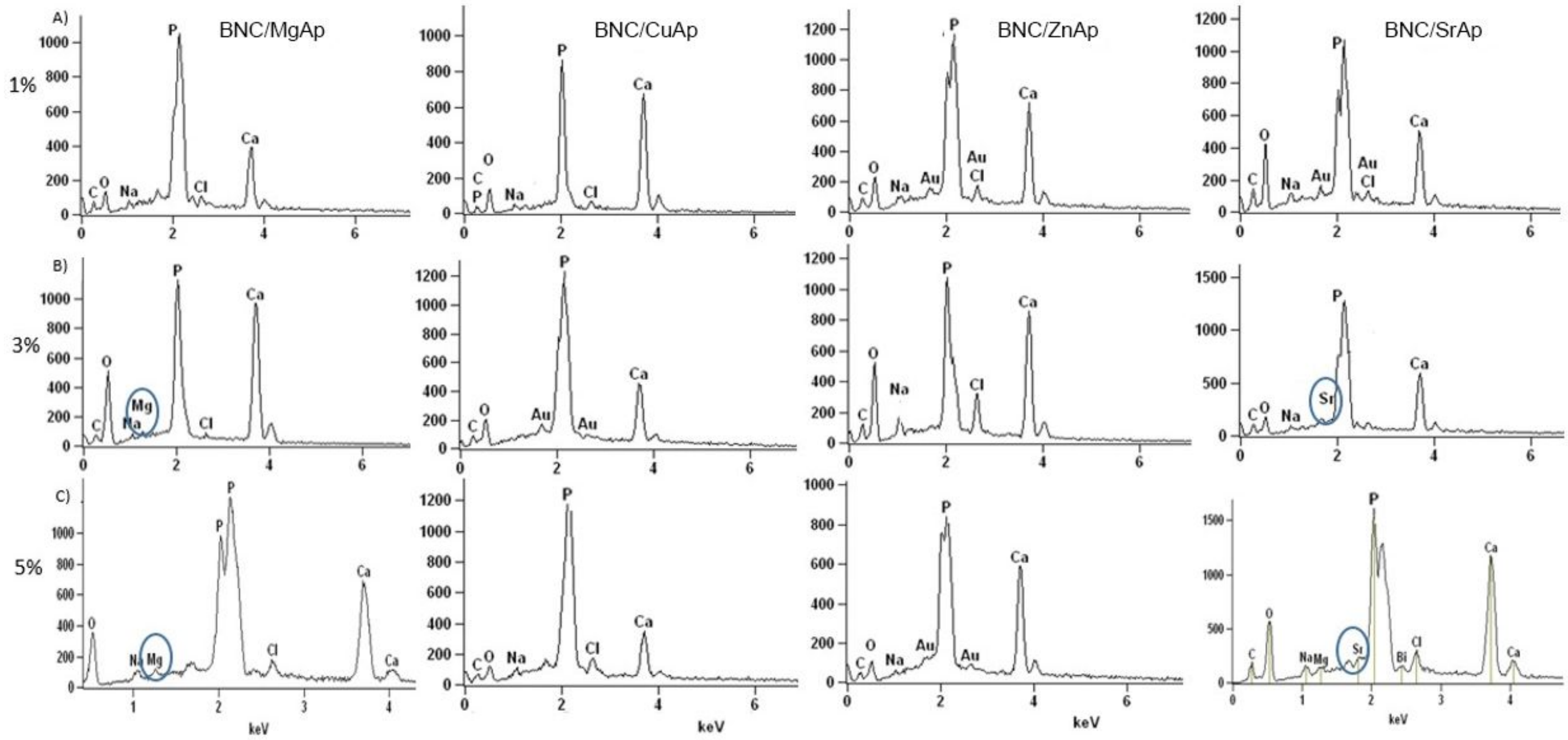

Figure S1: Biomaterials EDS BNC/MgAp; BNC/CuAp; BNC/ZnAp and BNC/SrAp.

Vibrational frequencies	BNC	BNC/HAp	BNC/ MgAp 1%	BNC/ MgAp 3%	BNC/ MgAp 5%	BNC/ CuAp 1%	BNC/ CuAp 3%	BNC/ CuAp 5%	BNC/ ZnAp 1%	BNC/ ZnAp 3%	BNC/ ZnAp 5%	BNC/ SrAp 1%	BNC/ SrAp 3%	BNC/ SrAp 5%	Ref.
OH stretching	3338	3344	3453	3349	3454	3351	3347	3450	3350	3347	3350	3350	3344	3341	42
Water OH bending	1635	1644	1639	1640	1641	1640	1638	1639	1641	1638	1648	1635	1639	1630	47
Stretching mode of CO_3^{2}	---	1415	1415	1415	1418	1418	1418	---	---	---	1418	1418	1416	1418	46,50
Antisymmetric bridge C–O–C stretching	1163	1142	---	---	---	1176	1196	1158	1158	1149	1162	---	1145	1147	44
C–O C-C stretching	1057	---	---	---	---	1078	---	---	1065	---	---	---	---	---	44
Asymmetric stretching vibration of P–O bond	---	1020	1020	1019	1020	1022	1017	1012	1021	1021	1023	1020	1020	1014	12
		958	963	961	960	962	960	957	961	962	960	963	960	960	45,46
Stretching mode of CO_3^{2}	873	871	871	872	868	872	871	869	872	872	869	871	871	871	46,47

Table S1. Data obtained from FTIR analysis for BNC membranes and biocomposites BNC/HAp (standard), BNC/MgAp, BNC/CuAp, BNC/ZnAp, and BNC/SrAp.

Samples	Mass $loss_1$ (%)	T_{peak2} (°C)	Mass $loss_2$ (%)	T_{peak3} (°C)	Mass $loss_3$ (%)	T_{peak4} (°C)	Mass $loss_4$ (%)	T_{peak5} (°C)	Mass $loss_5$ (%)	T_{peak6} (°C)	Mass $loss_6$ (%)	Residue (%)
**BNC/HAp**	3.78	119	1.02	232	3.28	320	24.46	412	5.51	763	1.60	58.23
**BNC/MgAp 1%**	3.08	122	1.76	220	4.09	315	28.78	435	5.65	791	1.95	52.14
**BNC/MgAp 3%**	3.33	126	2.91	-	-	330	26.72	443	5.02	702	0.56	60.79
**BNC/MgAp 5%**	2.95	123	2.39	-	-	332	25.73	444	4.77	-	-	62.82
**BNC/CuAp 1%**	1.78	125	2.81	-	-	317	27.54	437	6.50	754	0.66	59.67
**BNC/CuAp 3%**	1.50	126	3.38	-	-	319	26.27	428	5.20	735	0.86	62.29
**BNC/CuAp 5%**	2.14	126	2.36	234	7.34	314	19.15	444	5.15	856	10.00	51.96
**BNC/ZnAp 1%**	4.05	120	0.92	220	4.93	322	21.34	432	4.21	844	5.23	58.36
**BNC/ZnAp 3%**	3.20	119	1.53	224	9.73	330	17.40	452	3.31	885	13.98	50.16
**BNC/ZnAp 5%**	2.87	119	1.22	218	4.17	331	22.63	434	3.73	818	3.41	60.36
**BNC/SrAp 1%**	3.24	129	3.02	228	2.51	325	19.30	432	3.52	-	-	65.60
**BNC/SrAp 3%**	4.46	119	0.67	225	2.31	325	23.80	417	3.81	788	1.80	61.60
**BNC/SrAp 5%**	5.03	124	0.13	219	3.23	323	26.83	428	5.25	801	2.40	54.17

Table S2. Maximum degradation temperature (Tpeak) and percentage of mass loss (%) data obtained from the TG and DTG curves of BNC/HAp biocomposites (standard) and BNC/MgAp, BNC/CuAp, BNC/ZnAp and BNC/SrAp.
